# The Diagnosis of Yo-Yo Reflux with Dynamic Renal Scintigraphy in a Patient with Incomplete Ureteral Duplication

**DOI:** 10.4274/Mirt.71

**Published:** 2012-12-20

**Authors:** Özhan Özdoğan, Oğuz Ateş, Yeliz Kart, Feray Aras, Mustafa Olguner, Feza Akgür, Hatice Durak

**Affiliations:** 1 Dokuz Eylül University School of Medicine , Department of Nuclear Medicine, İzmir, Turkey; 2 Dokuz Eylül University School of Medicine , Department of Pediatric Surgery, İzmir, Turkey; 3 Celal Bayar University School of Medicine, Department of Nuclear Medicine, İzmir, Turkey

**Keywords:** 99mTc-MAG3, genitourinary abnormalities, vesico-ureteral reflux

## Abstract

The diagnosis of yo-yo reflux in patients with incomplete upper collecting system duplications is difficult. We report a case with recurrent urinary tract infections and ultrasonographically detected duplication in the left collecting system in which the presence of yo-yo reflux is demonstrated with dynamic renal scintigraphy.

**Conflict of interest:**None declared.

## INTRODUCTION

The embryological development of urinary system is complex and approximately 10% of the children are born with urinary anomalies (1). Duplication of the upper collecting system is one of the most common of these anomalies. Complete duplication is associated with ectopic ureter, ureterocele or vesicoureteral reflux. Therefore the diagnosis of symptomatic complete duplication is relatively easy. The incomplete duplication of the upper collecting system rarely causes urinary symptoms and usually does not carry clinically importance (2,3). However, reflux of the urine from one limb of the collecting system to the other limb, rather than down towards the bladder may cause urinary symptoms. This phenomenon is called yo-yo reflux (saddle reflux) and the diagnosis (to demonstrate the urine refluxing from one limb to the other limb) of yo-yo reflux is difficult. Chu et al has described a scintigraphic method for detection of yo-yo reflux (2,4). We present a case with yo-yo reflux in which the diagnosis has been made with similar scintigraphic method. We aimed to emphasize the diagnostic method to overwhelm the diagnostic difficulties encountered in yo-yo reflux. We present a case to emphasize the scintigraphic method to overwhelm the diagnostic difficulties encountered in yo-yo reflux. 

## CASE REPORT

A 6 year-old girl presented with a recurrent urinary tract infection (UTI) had an abdominal ultrasonography (US) which revealed an enlarged left duplex kidney with a mildly prominent lower moiety collecting system. Voiding cystourethrography was normal without post voiding residual urine. She was referred for the radionuclide renography to see the drainage pattern of the left kidney.

**Figure 1:**
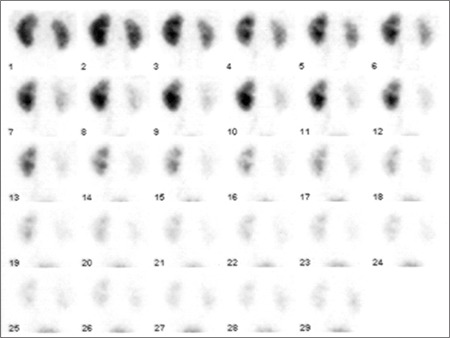
One minute dynamic planar images. The activity in lower moietystarts to increase by 8[ref:th]th[/ref] minute, becoming more prominent at 11[ref:th]th[/ref] minuteand than starts decreasing. Only a single left ureter is visible. Note the slightincrease in activity of upper moiety at 9-11 minute images which was interpretedas a minor reflux

**Figure 2:**
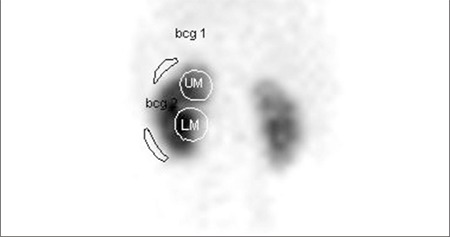
The summed function images (2-3 min) from posterior projectiondemonstrating the regions of interest drawn to upper moiety (UM), lowermoiety (LM) and background regions (bcg 1 and bcg 2 respectively)

**Figure 3:**
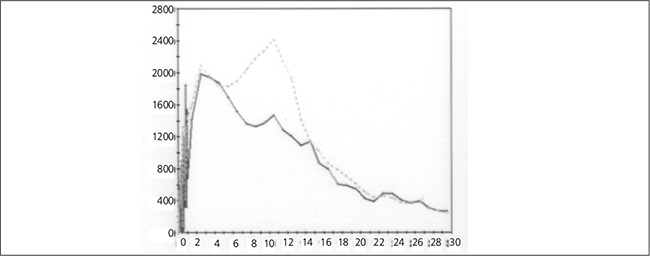
The time-activity curves of upper moiety (continuous trace) andlower moiety (dotted trace) of the left kidney. As the decline of activity inupper moiety was more prominent starting at fifth minute, a sharp inclineof activity on lower moiety was observed

Tc-99m mercaptoacetyltriglycine (MAG3) renal scintigraphy was performed according to F -15 protocol (5). Oral hydration (10 ml/kg) started 30 minutes before the study and intravenous furosemide (2 mg) was given 15 minutes before the intravenous administration of 1 mCi (37 MBq) of MAG3. Imaging was performed from posterior projection using GE XRT gamma camera equipped with a low energy parallel-hole general purpose collimator at 140 keV energy peak with a 20% symmetrical energy window. Dynamic acquisition at 64x64 matrix was acquired 1 second per frame for 1 minute for the renal perfusion and 60 seconds per frame for 29 minutes for the renal function. An Entegra (GE medical systems) workstation was used for image processing. The dynamic planar images of MAG3 renal scintigraphy showed more prominent activity in lower moiety than upper (Figure 1). The region of interests and the time activity curves are shown in Figure 2 and Figure 3, respectively. The time to peak parenchymal uptake of upper and lower moieties of left renal duplex system was at 2.5 minutes for each and at about the third minute both curves showed a gradual decline indicating the beginning of excretion and patency of the collecting systems (Figure 3). At the fifth minute, the slope of the activity curve of upper moiety increased, indicating a more rapid excretion followed by a focal rise of activity in lower moiety until the 10.5^th^ minute (Figure 3). Diuretic was injected before the study, intravenous line was washed with saline just after the injection of radiopharmaceutical and there was not any patient motion, so the increase of the activity in the lower moiety was explained by “yo-yo” reflux. The drainage of both moieties was complete until the end of the study excluding the presence of an obstruction.

The patient is well after the last episode of UTI that was treated with antibiotics, on regular follow up. Also a long term suppression antibiotherapy was given. Proper wiping technique following the toilet use and frequent urination were advised for conservative management. Surgical treatment was not indicated.

## LITERATURE REVIEW AND DISCUSSION

Yo-yo reflux is a rare clinical form of incomplete duplication of upper collecting system. Disorders in ureteric peristalsis at the site of ureteric fusion may cause yo-yo reflux. Another explanation of yo-yo reflux is the pressure gradient between two ureteric segments. The pressure of the lower moiety is generally higher than the upper moiety and therefore the urine generally refluxes from the lower moiety to the upper moiety (2,3). However this is not a rule as occurred in our case where the reflux was into the lower moiety.

There is no radiological diagnostic method that exactly shows urine refluxing from one moiety to another at incomplete duplex systems. The current method that we used has been recently described by Chu et al (4). They stated that the dynamic nature of the investigation and continuous monitoring makes the radionuclide renography an ideal test for the diagnosis of yo-yo reflux. The appropriately placed region of interests over the upper and the lower moiety of the renal duplex system give an opportunity to see changes in time activity curves of each moiety independently. 

The diagnosis of yo-yo reflux is difficult although the diagnosis of presence of collecting system duplication is not. Surgical treatment in yo-yo reflux should be considered in patients with significant dilatation or flank pain, accompanied by urinary tract infection (3). In the operation, the upper ureter is anastomosed to the lower pelvis, converting bifid ureter to bifid pelvis and excising the rest of the upper ureter (ureteropyelostomy) (6). Therefore, before making a decision for treatment, it is important to demonstrate refluxing urine from one moiety to the other in the dynamic renal scintigraphy. So the patients with a duplex kidney, one should consider yo-yo reflux if there is a simultaneous rise in the tracer activity in one moiety and a decline in the other. 

## References

[1] Rink CR, Adams MC, Mitchell ME, In Aschraft KW (1990). Ureteral abnormalities. Pediatric Urology.

[2] Gonzales E, Kelalis PP, King LR, Belman AB (1992). Anomalies of the Renal Pelvis and Ureter. Clinical Pediatric Urology.

[3] Smith P, Dunn M (1979). Duplication of the upper urinary tract. Ann R Coll Surg Engl.

[4] Chu WC, Chan KW, Metreweli C (2003). Scintigraphic detection of “yo-yo” phenomenon in incomplete ureteric duplication. Pediatr Radiol.

[5] Upsdell SM, Testa HJ, Lawson RS (1992). The F-15 diuresis renogram in suspected obstruction of the upper urinary tract. Br J Urol.

[6] Frank JD, O’Neill JA (1988). Ureteral duplication and ureteroceles. Pediatric surgery.

